# Multidrug-resistant *Acinetobacter baumannii* resists reactive oxygen species and survives in macrophages

**DOI:** 10.1038/s41598-019-53846-3

**Published:** 2019-11-25

**Authors:** Yoshinori Sato, Yuka Unno, Chizuru Miyazaki, Tsuneyuki Ubagai, Yasuo Ono

**Affiliations:** Department of Microbiology and Immunology, Teikyo University School of Medicine, 2-11-1 Kaga, Itabashi-ku, Tokyo 173-8605 Japan

**Keywords:** Bacteriology, Pathogens

## Abstract

We investigated the intracellular survival of multidrug-resistant *Acinetobacter baumannii* (MDRAB) clinical isolates in macrophages, after phagocytosis, to determine their virulence characteristics. After ATCC 19606 and 5 clinical isolates of MDRAB were phagocytosed by mouse and human macrophages, the bacterial count of MDRAB strains, R4 and R5, increased in the mouse macrophages, 24 hours after phagocytosis. Bacterial count of the strains, R1 and R2, was almost equal 4 and 24 hours after phagocytosis. Intracellular reactive oxygen species was detected in the macrophages after phagocytosis of these bacteria. Further, the strains R1, R2, R4, and R5 showed higher catalase activity than ATCC 19606. Additionally, strains R1, R4, and R5 grew more efficiently than ATCC 19606 in the presence of H_2_O_2_, whereas growth of strains R2 and R3 was marginally more than that of ATCC 19606 in the presence of H_2_O_2_. The MDRAB clinical isolates altered the expression of *TNF-α*, *IL-1β*, *IL-6*, and *MIP-2* mRNA induced in J774A.1 cells, 24 hours after phagocytosis. These results provide insights into the renewed virulence characteristics of MDRAB clinical isolates. Finally, tigecycline killed MDRAB phagocytosed by the macrophages more effectively than colistin, although colistin and tigecycline are both considered effective antibiotics for the treatment of MDRAB.

## Introduction

*Acinetobacter baumannii* is an important opportunistic pathogen, associated with nosocomial infections such as bacteraemia, pneumonia, meningitis, urinary tract infections, and wound infections^1,2^. The recent increase in outbreaks of multidrug resistant *A. baumannii* (MDRAB) worldwide is a cause for concern^3–5^. Additionally, *A. baumannii* is included among the 6 nosocomial pathogens: *Enterococcus faecium*, *Staphylococcus aureus*, *Klebsiella pneumoniae*, *Acinetobacter baumannii*, *Pseudomonas aeruginosa*, and *Enterobacter* spp. (ESKAPE) that acquire multidrug resistance and virulence^6,7^. Therefore, *A. baumannii*, especially MDRAB, has further gained importance as a human pathogen in the hospital environment.

Although *A. baumannii* is regarded as a low-virulence pathogen^8^, recent studies have clarified that *A. baumannii* shows several forms of pathogenicity such as biofilm formation, adherence, and invasion of lung epithelial cells^9–11^, host cell death^12–14^, and iron acquisition^15^. The pathogenicity of *A. baumannii* depends on various virulence factors, especially, the outer membrane proteins ‘Omps’ being vital in this respect^16^. Additionally, we have reported that clinical isolates of MDRAB show different levels of *omp* expression and exhibit different cell adherence capacities across strains^17^. Moreover, the clinical isolates show different degrees of biofilm formation in the presence of sub-minimum inhibitory concentrations of antibiotics^18^. These results suggest that *A. baumannii* is emerging as a highly pathogenic bacterium and that the characteristics of *A. baumannii* vary in different environmental stress conditions, such as multiple antimicrobial agents and host immune responses.

Phagocytic cells such as neutrophils and macrophages represent the first line of defence against invading bacterial pathogens in the host^19^. These cells ingest and eliminate microorganisms by the phagocytic process, which involves the formation of phagosome and subsequent maturation of this phagosome into a phagolysosome. A robust antimicrobial environment such as low pH, oxidative conditions, nutrient depletion, and antimicrobial peptides are provided within the phagolysosome^20^. The production of reactive oxygen species (ROS) within the phagolysosome is especially potent, leading to the destruction of microorganisms^20,21^. In fact, malfunctioning of ROS production in patients suffering from severe recurrent infections can lead to death in many cases^20,22,23^. In *A. baumannii* infections, the production of ROS or NO appears to contribute to bactericidal function of neutrophils and macrophages and plays a crucial role in host defence and survival^24,25^. As a defence mechanism, *S. aureus* expresses the enzymes super oxide dismutases and catalase that protect it against ROS and enable its survival within the phagolysosome^20^. Likewise, *A. baumannii* is a catalase-positive bacterium, where in, catalase is encoded by the *katE*/*katG* genes. Additionally, the universal stress protein UspA protects it against H_2_O_2_ stress^26,27^, suggesting that *A. baumannii* survives within phagolysosomes of macrophages through the degradation of H_2_O_2_ by its catalase activities. Although, the uptake of *A. baumannii* by alveolar macrophages and murine macrophage cell line J774A.1 has been explored^25^, few studies have focused on the intracellular survival of *A. baumannii* in macrophages because it is regarded as an extracellular pathogen.

We have previously reported that the renewed virulence characteristics of *A. baumannii* clinical isolates depend on its ability to adhere to human epithelial cells, and on the expression level of *omp* mRNAs^17^. These results might imply that since the clinical isolates of *A. baumannii* may have been exposed to various environmental stress conditions in the hospital, numerus virulence factors in the clinical isolates may have been modulated. Therefore, in this study, we have focused on the intracellular survival of MDRAB clinical isolates in macrophages, and their catalase activity. We have further evaluated the expression levels of ROS and proinflammatory cytokines in macrophages after phagocytosis with the aim of exploring the influence of intracellular bacteria on the functioning of macrophages. Finally, colistin and tigecycline, which are considered effective antibiotics for the treatment of MDRAB, have been evaluated for their ability to kill intracellular MDRAB clinical isolates within macrophages.

## Results

### MDRAB clinical isolates survive in macrophages

**Figure 1:**
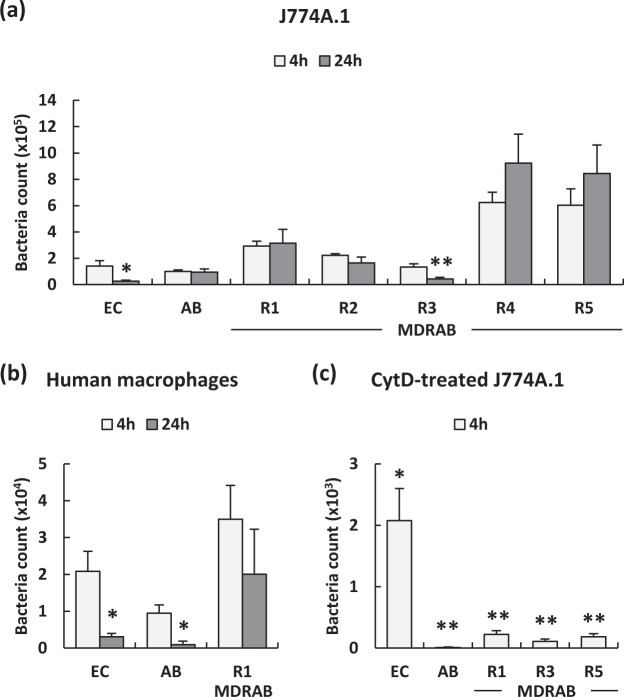
Intracellular survival of MDRAB clinical isolates phagocytosed by mouse and human macrophages. (**a**) Summarised results showing the bacterial count of *E. coli* (EC), ATCC 19606 (AB), and MDRAB clinical isolates in J774A.1 cells at 4 and 24 hours after phagocytosis. (**b**) Summarised results showing the bacterial count of EC, AB, and a representative MDRAB strain R1 in human macrophages at 4 and 24 hours after phagocytosis. (**c**) Summarised results showing the bacterial count of EC, AB, and 3 representative MDRAB strains R1, R3, and R5 in cytochalasin D (CytD)-treated J774A.1 cells at 4 hours after co-culture with these bacteria. Bar graph data are compiled from 2 independent experiments (n = 3 for each experiment), and represent the mean ± SEM. (**a**,**b**) Asterisks indicate statistically significant differences (**P < 0.01; *P < 0.05, 4 hours after phagocytosis *vs*. 24 hours after phagocytosis; Student’s *t*-test). (**c**) Asterisks indicate statistically significant differences (**P < 0.01; *P < 0.05, non-treated J774A.1 cells *vs*. CytD-treated J774A.1 cells; Student’s *t*-test).

Previous studies have shown that mouse macrophages can rapidly and efficiently phagocytose *A. baumannii in vitro* without the presence of antibody or complement opsonisation^25^. Therefore, we examined the MDRAB clinical isolate counts in J774A.1 and human macrophages at 4 and 24 hours after phagocytosis. As shown in Fig. 1a, *E. coli*, ATCC 19606, and 5 clinical isolates of MDRAB were detected in J774A.1 cells at 4 hours after phagocytosis. The bacterial count of *E. coli* and MDRAB strain R3, at 24 hours after phagocytosis, was significantly decreased compared with that at 4 hours after phagocytosis, whereas the bacterial counts of strains R4 and R5 at 24 hours after phagocytosis were increased compared with that at 4 hours after phagocytosis. Bacterial counts of the strains R1 and R2 were almost equal at 24 hours as well as 4 hours, after phagocytosis. Additionally, we examined the counts of *E. coli*, ATCC 19606, and a representative MDRAB strain, R1, in human macrophages at 4 and 24 hours after phagocytosis. As shown in Fig. 1b, the bacterial counts of *E. coli* and ATCC 19606 at 24 hours after phagocytosis were significantly decreased compared with that at 4 hours after phagocytosis, whereas a slight decrease in the count of strain R1 was observed at 24 hours compared with that at 4 hours, after phagocytosis. We next examined whether *A. baumannii* was phagocytosed by the macrophages or they invaded into the macrophages. J774A.1 cells were co-cultured with the bacteria in the presence of cytochalasin D (CytD), a potent inhibitor of micropinocytosis/phagocytosis. As shown in Fig. 1c, the bacterial counts of *E. coli*, ATCC 19606, and 3 representative MDRAB strains R1, R3, and R5 in CytD-treated J774A.1 cells were significantly lower than that in non-treated J774A.1 cells at 4 hours after phagocytosis. These results indicate that MDRAB clinical isolates were phagocytosed by the macrophages, following which, they managed to survive within the cells.

### MDRAB clinical isolates induce, but not alter, ROS production in macrophages

**Figure 2:**
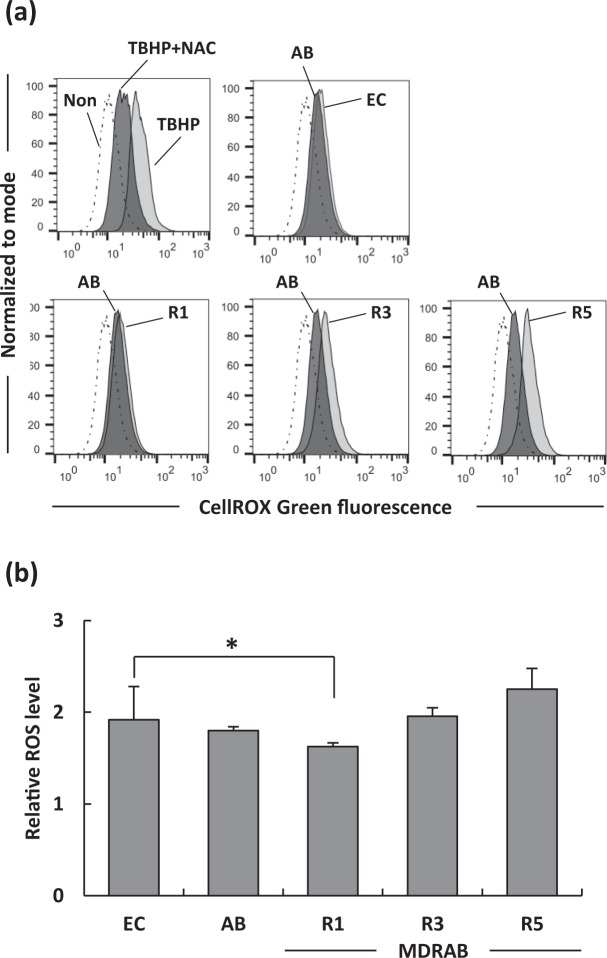
Intracellular ROS levels in mouse macrophages at 24 hours after phagocytosis. (**a**) Summarised results showing the intracellular ROS levels in J774A.1 cells at 24 hours after phagocytosis of *E. coli* (EC), ATCC 19606 (AB), and 3 representative MDRAB strains R1, R3, and R5. An oxidant, tert-butyl hydroperoxide (TBHP) (5 mM), was used as an inducer of ROS. N-acetyl cysteine (NAC) (5 mM) was used as an antioxidant. Mean fluorescence intensity (MFI) was measured by flow cytometry in untreated (dash line) and treated (black line) J774A.1 cells. (**b**) Relative MFIs are normalised to uninfected cells. Bar graph data are compiled from 2 independent experiments (n = 3 for each experiment), and represent the mean ± SEM. Asterisks indicate statistically significant differences (*P < 0.05; Student’s *t*-test).

On phagocytosis, the production of ROS in the phagolysosome plays a crucial role in destroying of microorganisms^20–23^. Therefore, we examined whether macrophages produced ROS in response to intracellular MDRAB. As shown in Fig. 2a, ROS production was detected in J774A.1 cells at 24 hours after phagocytosis of *E. coli*, ATCC 19606, and 3 representative MDRAB strains. However, the induction of ROS was of about the same level in *E. coli*, ATCC 19606, and MDRAB strains R3 and R5, whereas the ROS level in J774A.1 cells co-cultured with MDRAB strain R1 was slightly and significantly lower than that when J774A.1 cells were co-cultured with *E. coli* (Fig. 2b). These results suggest that macrophages produced ROS in response to intracellular MDRAB.

### MDRAB clinical isolates have high catalase activity

**Figure 3:**
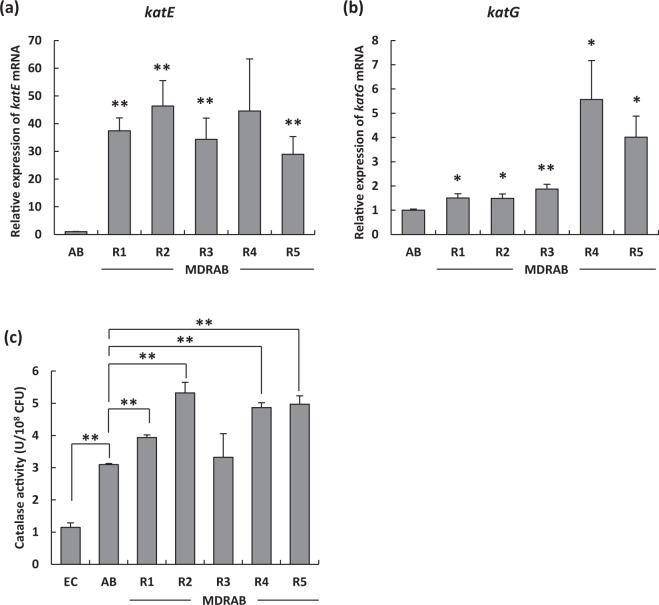
Catalase gene expression levels and activity of MDRAB clinical isolates. The mRNA expression levels of (**a**) *katE* and (**b**) *katG* in ATCC 19606 (AB) and MDRAB clinical isolates (**P < 0.01; *P < 0.05, AB *vs*. MDRAB; Student’s *t*-test). (**c**) Catalase activity of *E. coli* (EC), ATCC 19606 (AB), and MDRAB clinical isolates. Bar graph data represent the mean ± SEM and are compiled from 2 independent experiments (n = 3 for each experiment). Asterisks indicate statistically significant differences (**P < 0.01; *P < 0.05; Student’s *t*-test).

The results mentioned above suggest that macrophages produced ROS in response to intracellular MDRAB, however, could not eliminate intracellular MDRAB completely. As *A. baumannii* is a catalase-positive bacterium, we hypothesised that the catalase activity of MDRAB clinical isolates was upregulated. As shown in Fig. 3a, the expression of *katE* mRNA in MDRAB clinical isolates was significantly and substantially higher than that in ATCC 19606. Moreover, the expression of *katG* mRNA in strains R4 and R5 was significantly higher than that in ATCC 19606 (Fig. 3b). As shown in Fig. 3c, the catalase activity of ATCC 19606 was significantly higher than that of *E. coli*. Moreover, the catalase activity of the strains R1, R2, R4, and R5 was significantly higher than that of ATCC 19606, whereas catalase activity of strain R3 was almost equal to that of ATCC 19606. These results suggest that MDRAB clinical isolates exhibit upregulated catalase activity, which primarily depends on the expression level of the *katE* gene.

### MDRAB clinical isolates resist toxicity caused by hydrogen peroxide

**Figure 4:**
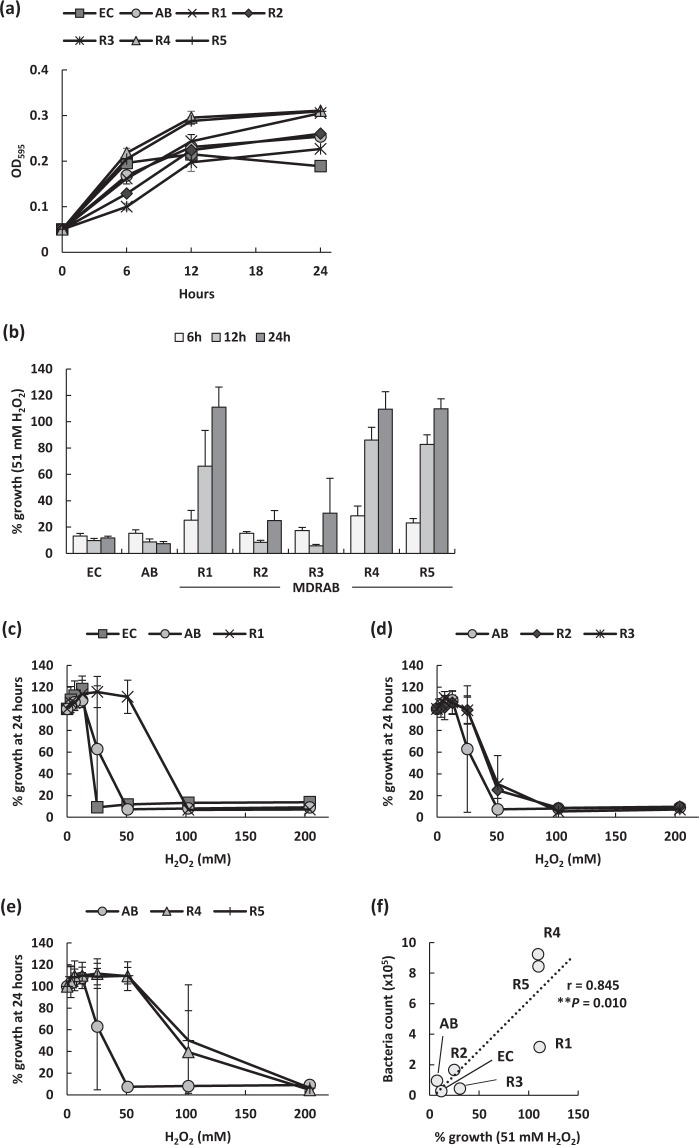
Bacterial growth of MDRAB clinical isolates in the presence of H_2_O_2_. (**a**) Bacterial growth of *E. coli* (EC), ATCC 19606 (AB), and MDRAB clinical isolates cultured in LB broth were evaluated. (**b**) Growth is represented as a percentage of the growth of the corresponding untreated strain. Summarised results showing the growth of *E. coli* (EC), ATCC 19606 (AB), and MDRAB clinical isolates in the presence of 51 mM H_2_O_2_ in 6, 12, and 24-hour cultures. (**c**) Growth is represented as a percentage of the growth of EC, AB, and strain R1 in the presence of H_2_O_2_ at the concentrations indicated on the x axis, in 24-hour culture. (**d**) Growth is represented as a percentage of the growth of AB and strains R2 and R3 in the presence of H_2_O_2_ at the concentrations indicated on the x axis in 24-hour culture. (**e**) Growth is represented as a percentage of the growth of AB and strains R4 and R5 in the presence of H_2_O_2_ at the concentrations indicated on the x axis in 24-hour culture. Data are compiled from 2 independent experiments (n = 3 for each experiment), and are shown as mean ± SEM. (**f**) Pearson correlation coefficient was calculated between the percentage of growth in the presence of 51 mM H_2_O_2_ and the bacterial count in J774A.1 cells at 24 hours after phagocytosis (Pearson correlation coefficient r = 0.845, *P* = 0.010). Each symbol represents a clinical isolate, AB, or EC.

ROS plays a crucial role in the rapid killing of *A. baumannii* ingested by phagocytes^24^. However, our study indicated that macrophages produced ROS in response to MDRAB clinical isolates, albeit, could not eliminate them. Additionally, MDRAB clinical isolates had high catalase activity (Fig. 3c). Therefore, to clarify the resistance of MDRAB clinical isolates to ROS, we evaluated the growth of MDRAB clinical isolates in a medium containing H_2_O_2_ *in vitro*. MDRAB clinical isolates showed very similar growth when cultured for 24 hours in the absence of H_2_O_2_ (Fig. 4a). However, *E. coli* and ATCC 19606 showed no growth when cultured for 24 hours in the presence of 51 mM H_2_O_2_, whereas MDRAB strains R2 and R3 showed little growth when cultured for the same time period (Fig. 4b). Further, MDRAB strains R1, R4, and R5 grew slightly at 6 hours of culturing in the presence of 51 mM H_2_O_2_, and these strains showed about 100% growth at 24 hours of culturing in the presence of 51 mM H_2_O_2_. Additionally, although *E. coli* and ATCC 19606 showed no growth when cultured for 24 hours in the presence of 51 mM H_2_O_2_, the MDRAB strain R1 grew in the presence of 51 mM H_2_O_2_ (Fig. 4c). Moreover, strains R2 and R3 showed little growth in the presence of 51 mM H_2_O_2_ (Fig. 4d), whereas strains R4 and R5 showed about 50% growth in the presence of 102 mM H_2_O_2_ (Fig. 4e). These results suggest that some MDRAB clinical isolates have the ability to survive under oxidative stress within the phagolysosome of macrophages. Pearson correlation analysis revealed that the intracellular bacterial count in the macrophages was positively and significantly correlated with the growth rate of bacteria in the presence of 51 mM H_2_O_2_ *in vitro* (r = 0.845, *P* = 0.010) (Fig. 4f). However, Pearson correlation analysis revealed that the catalase activity of these bacteria was not correlated with both, the intracellular bacterial count in macrophages as well as the growth rate of bacteria in the presence of 51 mM H_2_O_2_ *in vitro* (data not shown). This was consistent with the observation that the MDRAB strain R2 exhibited high catalase activity, however, could not survive in a medium containing H_2_O_2_.

### MDRAB clinical isolates alter the expression of proinflammatory cytokines in macrophages

**Figure 5:**
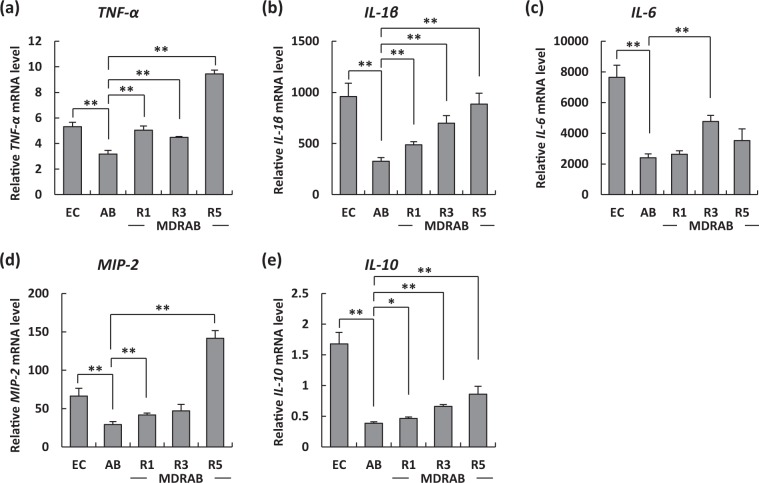
Proinflammatory cytokine expression levels in J774A.1 cells at 24 hours after phagocytosis. Summarised results showing the mRNA expression of proinflammatory cytokines in J774A.1 cells at 24 hours after phagocytosis of *E. coli* (EC), ATCC 19606 (AB), and 3 representative strains R1, R3, and R5. The mRNA levels of (**a**) *TNF-α*, (**b**) *IL-1β*, (**c**) *IL-6*, (**d**) *MIP-2*, and (**e**) *IL-10* were analysed by real-time PCR. Bar graph data represent the mean ± SEM and are compiled from 2 independent experiments (n = 3 for each experiment). Asterisks indicate statistically significant differences (**P < 0.01; *P < 0.05; Student’s *t*-test).

We have reported previously that MDRAB clinical isolates alter the expression of proinflammatory cytokines in human epithelial cells, suggesting that the clinical isolates had acquired renewed virulence characteristics^17^. Therefore, in this study, we evaluated the mRNA levels of proinflammatory cytokines in J774A.1 cells at 24 hours after phagocytosis of *E. coli*, ATCC 19606, and the MDRAB representative strains R1, R3, and R5. The expression of *TNF-α*, *IL-1β*, *IL-6*, and *MIP-2* mRNA was induced in J774A.1 cells at 24 hours after phagocytosis of the bacteria (Fig. 5a–d). The mRNA levels of *TNF-α* and *IL-1β* in J774A.1 cells at 24 hours after phagocytosis of ATCC 19606 were significantly lower than those after phagocytosis of *E. coli*, whereas those of the three representative MDRAB strains were significantly higher than those of ATCC 19606 (Fig. 5a,b). The mRNA level of *IL-6* in J774A.1 cells at 24 hours after phagocytosis of ATCC 19606 was significantly lower than that after phagocytosis of *E. coli*, whereas that of strain R3 was significantly higher than that of ATCC 19606 (Fig. 5c). The mRNA levels of *MIP-2* in J774A.1 cells at 24 hours after phagocytosis of ATCC 19606 were significantly lower than those after phagocytosis of *E. coli*, whereas those of strains R1 and R5 were significantly higher than those of ATCC 19606 (Fig. 5d). The expression of *IL-10* mRNA was lowest in J774A.1 cells at 24 hours after phagocytosis of ATCC 19606, and this expression was significantly higher in that of the three MDRAB strains, and highest in that of *E. coli* (Fig. 5e). These results suggest that MDRAB clinical isolates alter the expression of proinflammatory cytokines in macrophages through their virulence factors. However, Pearson correlation analysis revealed that the mRNA level of each proinflammatory cytokine was not significantly correlated with the intracellular bacterial count of the macrophages (data not shown).

### Tigecycline is an effective antibiotic for intracellular MDRAB

**Figure 6:**
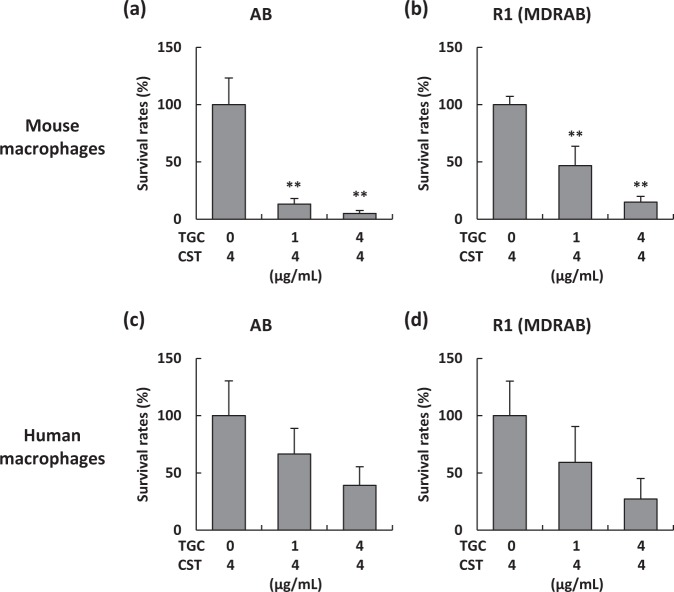
Antimicrobial effect of tigecycline on intracellular MDRAB. Summarised results showing the survival rates of intracellular bacteria in J774A.1 cells and human macrophages treated with colistin (CST) and tigecycline (TGC). (**a**) Survival rates of ATCC 19606 (AB) and (**b**) a representative strain R1 in J774A.1 cells (24-hour culture) treated with CST and TGC at the concentrations indicated on the on the x axis. (**c**) Survival rates of AB and (**d**) a representative strain R1 in human macrophages (24-hour culture) treated with CST and TGC at the concentrations indicated on the on the x axis. Bar graphs represent the mean ± SEM; data are compiled from 2 independent experiments (n = 3 for each experiment). Asterisks indicate statistically significant differences (**P < 0.01; One-way ANOVA).

Colistin and tigecycline are considered effective antibiotics for the treatment of MDRAB^28^. We evaluated whether these antibiotics were effective in killing intracellular MDRAB clinical isolates in the macrophages. Treatment of J774A.1 cells with a high concentration of colistin (50 μg/mL), used for the killing of extracellular MDRAB in the phagocytosis assay, did not successfully kill intracellular MDRAB (Figs. 1a and 6). These results indicate that colistin is not transported into the host cells. However, in mouse and human macrophages, the survival rates of ATCC 19606 and an MDRAB representative strain R1, were decreased in the presence of tigecycline in a dose-dependent manner (Fig. 6). These results suggest that tigecycline is an effective antibiotic for the killing of intracellular MDRAB.

## Discussion

*A. baumannii* has recently emerged as a major nosocomial pathogen^1,2^. Although, we have previously reported the virulence characteristics of *A. baumannii* clinical isolates in human epithelial cells^17^, the pathogenicity of *A. baumannii* clinical isolates within macrophages has remained elusive. Therefore, in the current study, we focused on the survival of intracellular MDRAB clinical isolates phagocytosed by macrophages, and the virulence factor responsible for the resistance to killing exhibited by this organism. Additionally, we evaluated the effect of antibiotics on intracellular MDRAB clinical isolates, commonly used for the treatment of MDRAB infections.

The role of macrophages in *A. baumannii* infection has been analysed in a previous study, and they are known to play a crucial role in early host defence, especially against respiratory *A. baumannii* infection^25^. Alveolar macrophages phagocytose *A. baumannii* and kill them with ROS and NO in the early stage of inflammatory responses in respiratory *A. baumannii* infections^24,25^. However, infection of mouse models with *A. baumannii* clinical isolates induced rapid bacterial replication in the lungs, significant extrapulmonary dissemination, and severe bacteremia by 24 hours of postintranasal inoculation, eventually leading to death of the animals, whereas infecting the animals with ATCC strains did not produce the same results^29^. In our previous study, we have reported that MDRAB clinical isolates express different levels of virulence factor *omps*, and exhibit high adherence capacity for human epithelial cells, compared with *A. baumannii* ATCC 19606^17^. These results suggest that clinical isolates of *A. baumannii* exhibited renewed virulence characteristics. Considering the above findings, in the current study, we examined the intracellular survival of MDRAB clinical isolates in macrophages *in vitro*. In fact, 2 of 5 clinical isolates survived and increased obviously in mouse macrophages for 24 hours after phagocytosis. These results suggest that MDRAB clinical isolates have acquired increased virulence capacity.

Considering the intracellular survival of clinical isolates of MDRAB in macrophages, it is likely that *A. baumannii* might evade the bactericidal action of ROS in phagolysosomes of macrophages. We evaluated the growth of MDRAB clinical isolates in a medium containing H_2_O_2_ *in vitro*, and clarified that MDRAB clinical isolates could grow more efficiently than ATCC 19606 in the presence of H_2_O_2_. We further clarified that MDRAB clinical isolates exhibited enhanced catalase activity as compared to the ATCC 19606 strain. *Acinetobacter* species have 4 predicted catalase genes, *katA*, *katE*, *katG*, and *katX*. Sun *et al*. reported that the capacities of *A. baumannii* and *A. nosocomialis* to degrade H_2_O_2_ are largely dependent on the *katE* gene and the resistance of both *A. baumannii* and *A. nosocomialis* to H_2_O_2_ is primarily determined by the *katG* gene, although *katE* also plays a minor role in H_2_O_2_ resistance^26^. In addition, the expression of *katE* mRNA in *A. baumannii* was drastically increased during the stationary growth phase, as compared to during exponential growth^26^. In the present study, we clarified that MDRAB clinical isolates have expressed high *katE* mRNA expression levels in the exponential growth phase. These results suggest that the clinical isolates have the capacity to degrade H_2_O_2_ in the exponential growth phase. Moreover, as the expression of *katG* mRNA in strains R4 and R5 was higher than that in other strains, we consider that, compared to the other strains, these two strains could grow in high concentrations of H_2_O_2_.

The universal stress protein UspA plays a crucial role in protecting *A. baumannii* from H_2_O_2_, low pH, and 2,4-DNP^27^. Moreover, a recent study has reported that OxyR (defined as a transcriptional regulator of H_2_O_2_ stress response)^30^ regulates the major H_2_O_2_-degrading enzymes, encoded by *katE* and *ahpF1*, (which encodes alkyl hydroperoxide reductase), in *A. baumannii*^31^. In the present study, the expression of *katE* mRNA in MDRAB clinical isolates was significantly higher than that in ATCC 19606, whereas the expression of *ahpF1* mRNA in MDRAB clinical isolates was slightly higher than that in ATCC 19606 (data not shown). Considering these results, novel transcription factors may regulate the expression of the *katE* gene. Additionally, the MDRAB strain R2 showed high catalase activity but could not survive in media containing H_2_O_2_. Further studies are required for in depth analysis of these clinical isolates.

Considering that *A. baumannii* has emerged as a major pathogenic species, this bacterium exhibits varying pathogenicity in different environments. Since *A. baumannii* clinical isolates may have been previously exposed to environmental stress conditions such as multiple antimicrobial agents, bactericidal substances in the serum, and host immune responses such as oxidative stress, they may have altered the expression of several genes in order to adapt to these stress conditions. Wright *et al*. found insertion sequence (IS) elements throughout the genome of *A. baumannii*, and these elements contributed to genome variation by interrupting genes or altering gene expression^32^. In addition, *A. baumannii* carrying an ISAba1 element upstream of the catalase-peroxidase gene *katG*, was selected by serial subculture in the presence of sub-inhibitory concentrations of H_2_O_2_^32^, suggesting that *A. baumannii* has the ability of adapting to oxidative stress. In the present study, although the expression of *katG* mRNA in strains R4 and R5 was significantly higher than that in ATCC 19606, no insertion sequence in the upstream region of the *katG* gene in MDRAB clinical isolates was found (data not shown). The DNA sequence upstream of the *katG* gene in MDRAB clinical isolates was the same as that in *A. baumannii* BJAB0868, BJAB07104, AC29, and AC30 clinical isolates^33,34^. As MDRAB clinical isolates exhibit enhanced catalase activity, further studies are required to determine novel transcription factors of *katG* as well as *katE* in these clinical isolates.

Colistin and tigecycline are considered effective antibiotics for the treatment of MDRAB^28^. Tigecycline acted synergistically with colistin, to exert antibacterial effects on *A. baumannii in vitro*^35^. However, cohort studies have reported that a combination of colistin and tigecycline against MDRAB, showed disappointing results^7,36^ and tigecycline-based therapy may not be the best option for treating MDRAB infection^37^. We clarified the antimicrobial effect of tigecycline on intracellular MDRAB that had survived in macrophages after phagocytosis *in vitro*. Our laboratory has previously reported a novel bacterial transport mechanism, where *A. baumannii* exploits human neutrophils by adhering to the cells and inducing IL-8 release for bacterial portage^38^. Moreover, this study clarified the intracellular survival of MDRAB clinical isolates in macrophages, and the results imply that *A. baumannii* spreads systemically by exploiting the macrophages. As colistin is an effective antibiotic for extracellular MDRAB, it may block the systemic spread of *A. baumannii* by targeting the neutrophils. Likewise, if tigecycline is an effective antibiotic for the killing of intracellular MDRAB, it may block the systemic spread of *A. baumannii* by targeting the macrophages. Further studies are required to clarify the role of colistin and tigecycline in controlling MDRAB infection.

In summary, we demonstrated that MDRAB clinical isolates acquired renewed virulence characteristics after phagocytosis by macrophages. High catalase production by MDRAB may impair the intracellular killing by macrophages and the consequent spread of *A. baumannii* infections. Further studies are required to understand the resistance mechanisms employed by MDRAB to evade killing by macrophages.

## Materials and Methods

All methods were carried out in accordance with relevant guidelines and regulations.

### Bacterial strains and growth condition

R1, R2, R3, R4, and R5 strains of *A. baumannii* were isolated from the Teikyo University hospital during an outbreak that occurred around 2010. The bacteria were isolated on CHROMagar™ *Acinetobacter* and incubated for 24 hours at 37 °C. As shown in Table 1, the R1 strain was isolated from the sputum of a patient with interstitial pneumonia. The R2 strain was isolated from a urine sample of a patient with malignant lymphoma and pneumonia. The R3 strain was isolated from the blood of a sepsis patient with myelodysplastic syndrome. The R4 strain was isolated from a stool sample of a patient with multiple myeloma and bacterial colonization. The R5 strain was isolated from the sputum of a patient with cardiovascular disease and bacterial colonization. The isolates were streaked onto blood agar plates and cultivated for 24 hours to obtain monoclonal colonies and identified as *A. baumannii* by DNA sequencing of a partial RNA polymerase β-subunit (*rpoB*) gene (La Scola *et al*., 2006). Additionally, the isolates were confirmed as non-clonal by pulsed-field gel electrophoresis (data not shown). After identification, these isolates were stored in glycerol stocks at −80 °C at the Department of Microbiology & Immunology, Teikyo University School of Medicine. Antimicrobial susceptibility testing was performed using 5 strains of *A. baumannii* based on the minimum inhibitory concentrations (MICs) of imipenem, amikacin, and ciprofloxacin. Against these 5 strains, the MICs of imipenem, amikacin, and ciprofloxacin were >8, 16, and 2 mg/L, respectively. These strains were thus identified as MDRAB strains. The *A. baumannii* ATCC19606 strain (AB) and the *Escherichia coli* ATCC 25922 strain (EC) were used as standard strains. The MIC of colistin against both ATCC 19606 and MDRAB strains, was determined as 2 mg/L. The MIC of tigecycline for ATCC 19606, and strains R1, R2, R4, and R5 was found to be 0.5 mg/L. The MIC of tigecycline for the R3 strain was 1 mg/L (Table 1). These bacteria were cultured on Luria-Bertani (LB) agar plates (Becton, Dickinson and Company, MD, USA) for 16 hours at 37 °C. Thereafter, the bacteria were suspended in RPMI-1640 Medium with L-glutamine and sodium (RPMI-1640) (Sigma-Aldrich, Tokyo, Japan), supplemented with 10% heat-inactivated fetal bovine serum (FBS) (Gibco, NY, USA) at a concentration of 2 × 10^7^ CFU/mL, with the concentration being adjusted via optical density (OD) measurements at 595 nm. The bacterial suspensions thus obtained were used for the phagocytosis assay.

**Table 1:** MDRAB clinical isolates used in the present study.

*A. baumannii*		MIC (μg/mL)		Isolation site	Reference
		CST	TGC		
ATCC 19606		2	0.5	Clinical isolate; type strain	ATCC
MDRAB (IPM > 8 mg/L, AMK = 6 mg/L, CPFX = 2 mg/L)	R1	2	0.5	The sputum of a patient with interstitial pneumonia	^17,18^
	R2	2	0.5	A urine sample of a patient with malignant lymphoma and pneumonia	^17,18^
	R3	2	1	The blood of a sepsis patient with myelodysplastic syndrome.	^17,18^
	R4	2	0.5	A stool sample of a patient with multiple myeloma and bacterial colonization	^17,18^
	R5	2	0.5	The sputum of a patient with cardiovascular disease and bacterial colonization	^17,18^

IPM, imipenem; AMK, amikacin; CPFX, ciprofloxacin; CST, colistin; TGC, tigecycline.

### Cell culture and phagocytosis assay

J774A.1 cells were purchased from the JCRB cell bank (Osaka, Japan), and maintained at 37 °C under 5% CO_2_ in RPMI-1640 medium supplemented with 10% FBS. J774A.1 cells were seeded at a concentration of 2 × 10^5^ cells/well in 24-well plates and cultured overnight. Prior to co-culture with *A. baumannii*, the J774A.1 cells were washed twice with PBS. The cells were then co-cultured at a multiplicity of infection of 100 bacteria per cell at 37 °C under 5% CO_2_ for 2 hours. In order to determine the intracellular viable bacteria at 4 and 24 hours, after 2 hours of co-culture, cells were washed 3 times with PBS, following which they were maintained in RPMI-1640 medium, supplemented with 10% FBS and 50 mg/mL colistin to kill the extracellular bacteria. To inhibit the phagocytosis of J774A.1 cells, they were pre-incubated for 1 hour with cytochalasin D (CytD) (FUJIFILM Wako Pure Chemical Corporation, Osaka, Japan) (5 μg/mL) before co-culture with bacteria and were kept with CytD during co-culture with bacteria. To analyse the number of intracellular bacteria, J774A.1 cells were washed thoroughly 3 times with PBS after co-culture. The bacteria were harvested after lysing the J774A.1 cells by adding sterile distilled water (1 mL) to each well; bacterial count was confirmed by the growth of serial dilutions of the bacterial suspension on LB agar in terms of CFUs after 24 hours of incubation at 37 °C.

### Differentiation of human macrophages

Human peripheral blood mononuclear cells (PBMC) were isolated from the peripheral venous blood of healthy volunteers. Briefly, whole blood (20 mL) was mixed with 7 mL of a 6% dextran solution and 15 mL of HBSS and allowed to stand for 30 minutes at 25 °C until stratification occurred. The upper leukocyte-rich plasma layer was transferred to a new tube containing endotoxin-free Ficoll-Paque PLUS gradient (GE Healthcare Japan, Tokyo, Japan) and was centrifuged (500 g, 30 min, 25 °C). The cell layer was harvested and subsequently, the cells were resuspended at 100 million PBMC per mL in Monocyte Attachment Medium (Promocell, Heidelberg, Germany). The cells were cultured for 10 days as per the manufacturer’s protocol and used for the phagocytosis assay, as described above. The protocol was approved by the Ethical Review Committee at the Teikyo University School of Medicine (no. 15-192-2). All participants gave a written informed consent prior to their inclusion in the study.

### RNA extraction and quantitative real-time polymerase chain reaction (qPCR)

To analyse the expression of proinflammatory cytokines, total RNA was extracted from J774A.1 cells after 24 hours of co-culture with bacteria, using an RNeasy Plus Mini kit (Qiagen, Tokyo, Japan). Total RNA of *A. baumannii* cultured in LB broth for 6 hours at 37 °C was extracted using an RNeasy Protect Bacteria mini kit (Qiagen). Harvested RNA samples were quantified using the NanoDrop spectrophotometer (Thermo Fisher Scientific, MA, USA). Total RNA was reverse-transcribed to cDNA using PrimeScript™ 1^st^ strand cDNA Synthesis Kit (Takara Bio, Shiga, Japan). To analyse the mRNA levels of all genes, cDNA was amplified using the SYBR Green PCR Master Mix (Thermo Fisher Scientific) with consensus primers for detecting *rpoB*, *katE*, *katG*, mouse *GAPDH*, mouse *TNF-α*, mouse *IL-1β*, mouse *IL-6*, mouse *MIP-2*, and mouse *IL-10*. The primer sequences are listed in Table 2. *rpoB* was used as an internal control for the quantification of *katE* and *katG*. Mouse *GAPDH* was used as an internal control for the quantification of mouse *TNF-α*, mouse *IL-1β*, mouse *IL-6*, mouse *MIP-2*, and mouse *IL-10*. Real-time PCR was performed as follows: 40 cycles of denaturation at 95 °C for 15 seconds, annealing at 60 °C for 30 seconds, and extension at 72 °C for 1 minute. The amplified PCR products were quantitatively monitored using a StepOne Real-Time PCR System (Applied Biosystems, CA, USA). Fold changes in the expression level of each gene were calculated by the 2^−ΔΔCt^ method using *rpoB* or mouse *GAPDH* gene as an internal control. The relative expression of each gene was evaluated relative to the control sample (ATCC 19606 or uninfected cells), which was assigned a value of 1 arbitrary unit.

**Table 2:** Primers used for real-time PCR.

Gene	Sequence	Reference
*katE*	F: AACTTTGACTTCGATTTGCTGGA R: TGTATGAAAATAGACGGGCTTGT	^26^
*katG*	F: GGCGATGAAAAAGAATGGTTA R: ATTTCTTCATCATCCATTGCC	^26^
*rpoB*	F: ATGCCGCCTGAAAAAGTAAC R: CGAGCGCCTACTGGAATTA	^17,18^
*Mouse TNF-α*	F: ATGATCCGCGACGTGGAA R: CTGCCACAAGCAGGAATGAG	^39^
*Mouse IL-1β*	F: CGCAGCAGCACATCAACAAGAGC R: TGTCCTCATCCTGGAAGGTCCACG	^40^
*Mouse IL-6*	F: CCAGAGATACAAAGAAATGATGG R: ACTCCAGAAGACCAGAGGAAA	^40^
*Mouse MIP-2*	F: ACCAACCACCAGGCTACAG R: GCGTCACACTCAAGCTCT	^41^
*Mouse IL-10*	F: CAGAGCCACATGCTCCTAGA R: GTCCAGCTGGTCCTTTGTTT	^40^
*Mouse GAPDH*	F: CTTCACCACCATGGAGAAGGC R: GGCATGGACTGTGGTCATGAG	^40^

F, forward primer; R, reverse primer.

### ROS production level

ROS production level in J774A.1 cells cultured for 24 hours after phagocytosis was detected by CellROX^®^ Green Flow Cytometry Assay Kit (Thermo Fisher Scientific). Briefly, J774A.1 cells cultured for 24 hours after phagocytosis, were washed with PBS and incubated in RPMI-1640 medium supplemented with 10% FBS and CellROX^®^ reagent at 37 °C for 30 min, as per the manufacturer’s protocols. The cells were harvested and washed with PBS, and subsequently fixed with BD Cytofix™ Fixation Buffer (BD Biosciences, San Jose, CA). The stained cells were analysed by using a FACSCanto II flow cytometer (BD Biosciences) equipped with a FACS Diva software. All flow cytometric data were analysed using FlowJo software (BD Biosciences). ROS levels were measured as the Median Fluorescence Intensity (MFI) for CellROX^®^ Green marker. The relative ROS level was evaluated relative to the control sample (uninfected cells), which was assigned a value of 1 arbitrary unit.

### Catalase activity

Catalase activity of the bacteria was measured using Catalase Colorimetric Activity Kit (Arbor Assays, MI, USA) by following the manufacturer’s protocols. Briefly, the bacterial strains were cultured on LB agar plates (Becton, Dickinson and Company, MD, USA) for 16 hours at 37 °C, after which, they were suspended in H_2_O at a concentration of 2.5 × 10^7^ cells/mL, with the concentration being adjusted via OD measurements at 595 nm. Thereafter, the bacterial cell suspension was used for catalase activity assay.

### H_2_O_2_ growth assay

H_2_O_2_ growth assay was conducted according to a previous study^31^. Bacterial strains were cultured on LB agar plates (Becton, Dickinson and Company, MD, USA) for 16 hours at 37 °C, after which they were suspended in LB broth at a concentration of OD_595_ = 0.05. The bacterial cell suspensions were subcultured for 1 hour prior to treatment with H_2_O_2_ (30%; Nacalai tesque, Kyoto, Japan) at the concentrations indicated in the figures. All growth assays were carried out in 96-well plates in a 100 μL volume, and growth was measured by OD_595_.

### Correlation analysis and statistics

Quantitative results were combined from 2 independent experiments (n = 6) and presented as mean ± standard error of the mean (SEM). Comparisons of numerical data were performed using Student’s *t*-test or one-way analysis of variance (ANOVA), followed by the Dunnett’s multiple comparison test. Pearson correlation analysis was used to compare the intracellular bacterial count in macrophages and % growth of bacteria in the presence of 51 mM H_2_O_2_. In all analyses, a 2-tailed probability of < 5% (i.e. *p < 0.05) was considered statistically significant.
